# Regulatory Mechanism of Intestinal Stem Cells Based on Hippo Pathway and Signaling Crosstalk in Chicken

**DOI:** 10.3390/ijms26115067

**Published:** 2025-05-24

**Authors:** Tao Quan, Ran Li, Yaoxing Chen, Ting Gao

**Affiliations:** College of Veterinary Medicine, China Agricultural University, Beijing 100083, China; s20243051072@cau.edu.cn (T.Q.); 18246269672@163.com (R.L.); yxchen@cau.edu.cn (Y.C.)

**Keywords:** intestinal stem cells, intestinal mucosa, Hippo pathway, intestinal epithelial cell

## Abstract

Recently, there has been a gradual increase in the demand for chicken and eggs. The gut, as the vital place of nutrient digestion and absorption, is highly associated with the development of livestock and poultry and the quality of meat, eggs, and milk. Intestinal stem cells, as an important source of intestinal cell proliferation and renewal, exert a vital effect on repairing injured intestinal epithelial cells and keeping homeostasis. Intestinal stem cell-regulated intestinal epithelial balance is closely controlled and modulated by interlinked developmental loops that maintain cell proliferation and differentiation processes in balance. Some conservative signaling pathways, including the Wnt, Notch, hedgehog, and bone morphogenetic protein (BMP) loops, have been proved to modulate intestinal health in poultry. Meanwhile, studies have revealed the importance of the Hippo pathway in gastrointestinal tract physiology by regulating intestinal stem cells. Moreover, crosstalk between Hippo and other signaling pathways provides tight, yet versatile, regulation of tissue homeostasis. In this review, we summarize studies on the role of the Hippo pathway in the intestine in these physiological processes and the underlying mechanisms responsible via interacting with these signaling pathways and discuss future research directions and potential therapeutic strategies targeting Hippo signaling in intestinal disease. A comprehensive understanding of how these signaling pathways regulate stem cell proliferation, differentiation, and self-renewal will help to understand the regulation of intestinal homeostasis. In addition, it has the capacity for creative ways to govern intestinal damage, enteritis, and associated disorders induced by different factors.

## 1. Introduction

**Figure 1:**
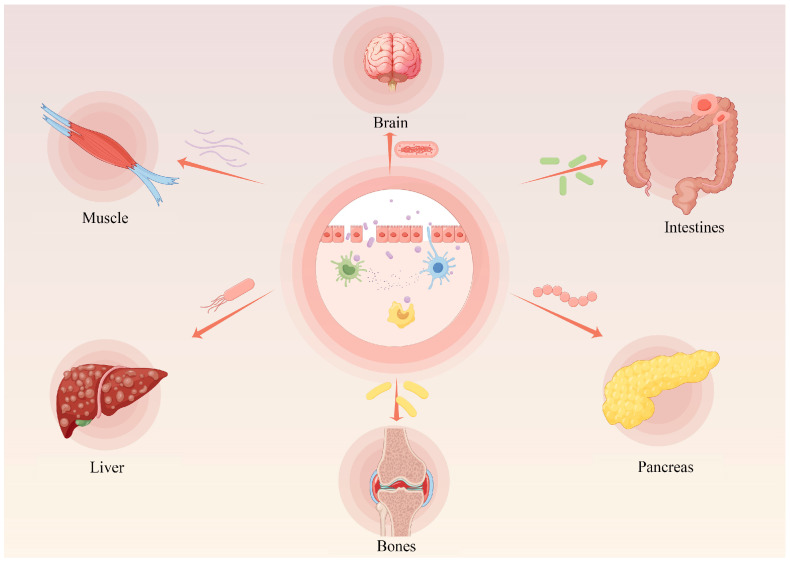
Intestinal health is closely related to the homeostasis of other organs in the body. Intestinal health is not only about the gut itself, but also about the transmission of gut microbiota and its metabolites through the bloodstream, which in turn affects the health status of other organs in the body. When the intestinal mucosal barrier is damaged and the intestinal microbiota is disturbed, it may be closely related to multi-organ health including brain, muscle, liver, bones and pancreas etc.

In recent years, there has been a gradual increase in the demand for chicken and eggs; the production of chicken meat will up-regulate from 117 million tons to 132 million tons in 2026 [1]. Several studies suggested that intestinal health is highly associated with metabolite state [2], immunocompetence homeostasis [3], host development [4], and general health [5], suggesting that intestinal homeostasis is a vital factor of homeostasis. Poultry digestive tract discomfort will not only affect the digestion and absorption of nutrients, but also undermine the health of the body, directly causing economic losses to the livestock and poultry industry. In contrast, in mammals, an imbalance of intestinal homeostasis not only induces a variety of intestinal-related disorders, including Crohn’s disease, ulcerative colitis, etc., but also has the potential to induce lesions in other organs and tissues, such as Alzheimer’s disease, type I diabetes, muscular dystrophy, and cancers, etc. [6] (Figure 1). Thus, maintaining intestinal homeostasis is essential for livestock and host homeostasis.

Anatomy divides the gut into the small intestines at the front and the large intestine at the posterior end, and the large intestine is dominated by the cecum in poultry. The small intestines are the main position of nutrient digestion and absorption in chickens. Nutrient transporters and digestive enzymes contained in the small intestinal pouch can resist pathogen invasion by secreting hormones, glycoproteins, and defensins [7,8]. The small intestinal epithelium folds internally to form the intestinal crypts and protrudes externally to produce intestinal villi, which are straight exposed to the intestinal lumen [9]. The intestinal epithelial lining is derived from the embryonic endoderm. Stem cells located in the intestinal crypt develop specialized types of epithelial cells that constitute the mature intestinal epithelium, and the crypt ecological niche provides essential signals and regulators for this process. These mature intestinal epithelial cells are distributed bottom-up along the intestinal villi, exert a vital effect on absorptive and secretory [10,11]. In chickens, proliferating cells are also distributed on the intestinal villi, which may be due to the kinetics of cell production from humans [12]. The chicken are widely used to study vertebrate embryonic development. While few studies have reported the molecular basis of epithelial cell development and differentiation in the chicken small intestine. Despite the limited knowledge of the characteristics of the chicken intestinal epithelium, recent researches have suggested that the chicken small intestinal epithelium has a number of similarities, although it is markedly different and uniquely characterized from that of humans and mice. Therefore, it is crucial to understand specific characteristics of chicken small intestinal epithelial cells.

Intestinal epithelial health is highly modulated by interrelated developmental loops and maintain cell proliferation and differentiation processes in balance. Many conserved signaling loops, including Wnt, Notch, hedgehog and bone morphogenetic protein (BMP) loops, have been indicated to modulate gut health and regeneration in mammals [13]. Recognizing how the Hippo pathway interacts with these signaling pathways could facilitate the understanding of the phenotypes that emerge in mouse models of Hippo signaling malfunctions and would help in the development of new therapeutic measures.

The Hippo pathway plays an important role in the regulation of cell proliferation, differentiation and death, and directly determines cell fate, such as tissue homeostasis and organ size [14,15], and disruption of this pathway can trigger the development of a variety of malignant diseases [16,17]. Several studies have demonstrated that the Hippo pathway plays a key role in gastrointestinal health [18]. Intestinal regeneration is co-regulated by multiple signaling pathways, and there is also a correlation between these different signaling pathways. The role of the Hippo pathway in intestinal regeneration has been reported. Meanwhile, interactions between the Hippo pathway and signaling pathways, including Wnt, Notch, and others, also play a key role in regulating intestinal homeostasis. Here, we outline how the Hippo pathway is involved in intestinal physiological functions and the potential mechanisms by which it plays a role, and accordingly summarize possible future research directions and propose therapeutic options in the treatment of intestinal diseases.

The paper reviews the development and growth of the chicken intestine and compares the cellular features and signaling loops, including the Notch and Wnt signaling pathways that control small intestinal epithelial cell differentiation, between chicken and mammals. Evidence suggests that the chicken intestinal epithelium has unique cellular structures and proliferative zones. The establishment of an in vitro cell culture model of chicken will offer a new artifact for exploring the molecular modulation of chicken intestinal development and progression and may also result in novel ideas for enhancing small intestinal health and nutrition in the poultry industry.

## 2. The Development of Chicken Small Intestine

The small intestines of the chicken consist of intestinal tubes, which are derived from the internal folding of the foregut and caudal intestinal entrances of the embryonic endoderm [19]. Intestinal tube formation is accomplished and modulated by diverse signals caused by Sonic hedgehog or Wnt [20,21], when the yolk stalk connecting the yolk sac is only lightly open around embryonic day e5 [22]. Between e5 and hatching day, the length of the small intestine increases [23]. Peristalsis within the chick small intestine was also observed at e5, suggesting the emergence of mechanical intestinal function during early embryogenesis [24]. By contrast, the expression of nutrient transporters, mineral transporters, and digestive enzymes is detected only at the final stage of embryonic development, while physiological structures such as the intestinal villi and crypts are formed on the day of hatching [25]. Mature and functional epithelial cells of the chicken small intestine, including goblet cells and intestinal epithelial cells, have been histologically examined by e18 [26]. As chickens are precocious species, the intestines are thoroughly mature at the end of embryogenesis and they are able to feed independently promptly after hatching. Thus, the production of the crypt–villus framework and functional epithelial cells is a precondition before hatching.

## 3. Cellular Proliferation in the Chicken Intestinal Epithelium

**Figure 2:**
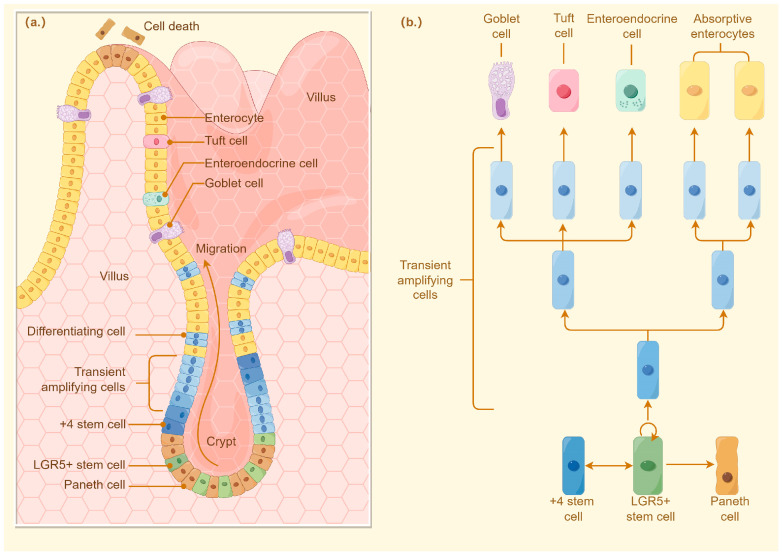
Proliferation and differentiation of intestinal stem cells. ISC is capable of differentiating into progenitor cells, and these newly formed cells proliferate and differentiate along the crypt–villus axis of the small intestine and colon. Lgr5 is an important marker of active ISC identified to generate differentiated epithelium cell types over a long period of time. In addition, another population of quiescent “reserve” ISC is located at the so-called ‘+4’ position. In the small intestine, ISC can differentiate into five major cell types, including enterocytes, goblet cells, enteroendocrine cells, tuft cells, and Paneth cells, but only differentiate into three major cell types in the colon. (**a**) Different cell types in the intestinal epithelium; (**b**) Differentiation of stem cells into different types of intestinal epithelial cell processes.

The intestinal crypt, first identified by Jonathan Lieberkühn, is a position where ISCs have the proliferative capacity to proliferate and differentiate, thereby maintaining homeostasis of the intestinal epithelium. For mammals, the proliferation and differentiation of intestinal stem cells are controlled by complex cellular cascades of signals that are regulated by Paneth cells in the crypt or by intercellular communication between stromal cells around the crypt [27]. Differentiation of intestinal stem cells initially forms transport amplification (TA). Proliferating cells rapidly propagate epithelial cell populations and migrate into the intestinal crypts along the villi generating ultimately differentiated cells (enterocytes, cisternae, enteroendocrine cells) or deeper into the crypts differentiating into Paneth cells [28]. Thus, the proliferative zone of mammalian intestinal epithelial cells is located within the crypt (Figure 2).

However, in addition to the crypts, chicken epithelial cells also proliferated extended to the villi, a conclusion reached by measuring the rate of uptake of thymidine at 3 hr. Immunohistochemical tests observed that 5-bromo-2-deoxyuridine (BrdU) and proliferating cell nuclear antigen (PCNA) were also localized to the villi in chicken small intestine. However, most of the proliferative activity of intestinal stem cells still occurred in the crypts [29]. Bar-Shira and Friedman also observed a small number of chromosome-concentrated proliferating columnar cells in chicken duodenal villi by hematoxylin and eosin staining. They also observed that lysozyme-secreting cells, probably chicken Paneth cells, were distributed in both crypts and villi. Considering that Paneth cells play an important role in the proliferation and differentiation of intestinal stem cells, it has been suggested that the proliferating cells found in chicken intestinal villi may be either intestinal stem cells or TA proliferating cells [30]. Yet another study identified cells expressing the leucine-rich repeat-containing G protein-coupled receptor 5 (*Lgr5*) (leucine-rich G protein-coupled receptor 5) and olfactory toxin 4 (*Olfm4*) genes in the crypts of chicken small intestine [31]. Therefore, chicken ISCs may be confined to the crypts, and the proliferating cells observed extend the villi may be delayed-differentiated TA cells. In addition, partially differentiated TA cells may also be present in the region above Olfm4-positive stem cells in the crypts and below PepT1 (oligopeptide transporter 1)-positive enterocytes extend the villi [32].

## 4. The Importance of Intestinal Stem Cells

Under normal physiological balanced conditions, our intestinal mucosa, immune system, intestinal microorganisms, nutrients, and metabolites interact with each other to form a dynamic equilibrium. Disturbances in the intestinal microenvironment or endogenous pathogens can disrupt the homeostatic balance and lead to intestinal epithelial damage and lesions. Therefore, ISC must actively play the functions of regeneration and repair to replenish the damaged and lost cells. In this process, ISC need to precisely regulate the homeostasis between proliferation and differentiation. When the self-renewal capacity of ISC is unregulated and over-enhanced, it may cause the growth of ISCs and their progeny to be unregulated; on the other hand, when the differentiation process of ISC is inhibited, or impaired, the proliferative capacity of the cells may also become stronger. Both of these conditions can lead to imbalances in gut homeostasis in poultry and thus induce gut-related diseases.

On the contrary, when the self-renewal ability of ISC is weakened or impaired, the stem cells age prematurely and cannot be replenished with new stem cells, leading to a decrease of the ISCs number, which results in a down-regulation in the functional cells number in tissues and organs due to the lack of replenished cells; in addition, if the differentiation of the ISCs is impaired, it also results in a down-regulation of functional cells number in tissues and organs. When the number of functional cells cannot meet the wear and tear of tissues and organs, it will lead to incomplete renewal and repair of tissues and organs, which will lead to tissue and organ failure. A recent study has revealed the mechanism by which genomic instability in ISC triggers stem cell necrosis, leading to spontaneous intestinal inflammation. The research suggested that targeting ISC necrosis may be an approach to treat severe intestinal inflammation. ISC, as important players in intestinal renewal and maintenance, are highly associated with the pathogenesis of several disorders. More and more studies are now linking intestinal diseases to stem cell defects, providing additional dimensions and possibilities for us to be able to better understand and treat intestinal diseases.

## 5. Homeostatic Maintenance of Intestinal Stem Cells

**Figure 3:**
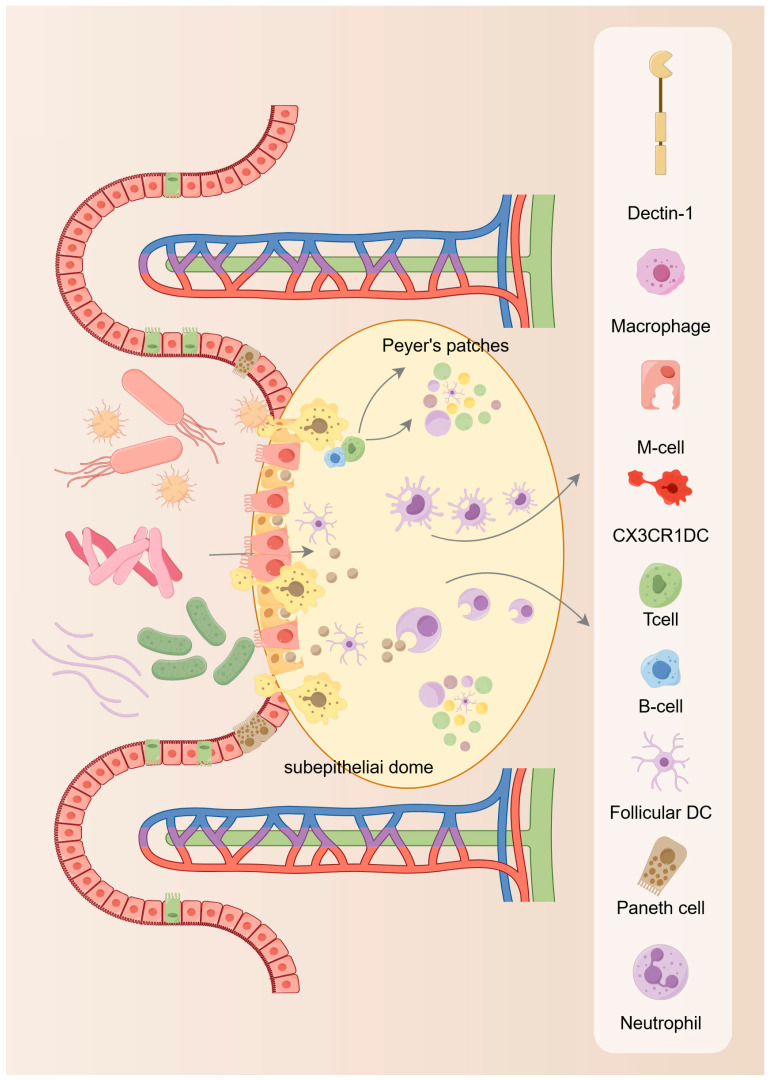
Maintenance of intestinal homeostasis. The intestinal tract plays a vital role in both digestion and immunity, making its equilibrium crucial for overall health. This equilibrium relies on the dynamic interplay between various intestinal cells, including intestinal epithelial cells, immune cells, crypt stem cells, and intestinal microbiota. Intestinal epithelial cells play a pivotal role in protecting and regulating the gut. They form vital barriers, modulate immune responses, and engage in pathogen defense and cytokine secretion. Moreover, they supervise the regulation of ISC. Immune cells actively influence the immune response through the phagocytosis of pathogens and the release of cytokines.

The proliferation and differentiation of ISC from progenitor cells into many specialized typologies of epithelial cells allows the intestinal epithelium to be continuously renewed to maintain intestinal homeostasis [33,34]. In this process, the ISC niche offers the microecology for proliferation and differentiation. In the ISC niche, the presence of many different types of cells provides different signals for the normal functioning of ISC to ensure that these ISC are able to differentiate into normal intestinal epithelium and to prevent the progress of tumor cells. The current development of gut tissue has greatly improved comprehension of the formation of the ecological niche of ISC [35]. Existing studies have identified two main components of the ISC ecosystem: the extracellular matrix (ECM) and the cellular microecology, which encompasses all resident cells embedded in the outer matrix, such as immune cells, fibroblasts, smooth muscle cells, etc. These cells can nourish ISC by secreting different matrix components and growth factors to promote their renewal and differentiation [36,37]. From the available findings, cells in the gut microecology, including Paneth cells and mesenchymal telangiectasia, can offer growth factors including Wnt and Notch ligands or epidermal growth factor (EGF) to promote epithelial cell proliferation and self-renewal [38]. However, when the renewal process of the intestinal epithelium is impaired, such as ecological dysregulation or immune dysfunction, including necrotizing enterocolitis, can occur (Figure 3).

### 5.1. Intestinal Mesenchymal Cells

Intestinal mesenchymal stromal cells, including fibroblasts, muscles, fibroblasts, endothelial cells, and smooth muscle cells, not only provide structured supports for ISC, but also secrete Wnt ligands and bone morphogenetic proteins (BMP) antagonists to regulate ISC activity [39]. New research indicated that a down-regulation of the Foxl1+ mesenchymal stem cells (MSCs) number resulted in a decrease in the secretion of Wnt signaling, which induced dysfunction of ISC and ultimately led to intestinal inactivation in mice. Another related study showed that Wnt2B can also be derived from Gli1+ MSCs, which are also critical for the functional maintenance of ISC. Various types of MSCs originate from ISC ecological niches (e.g., Wnt2B, R-spondin 1, Gremlin 1, and CD34+), which is an important feature of ISC [40]. The above studies establish the relevance of MSCs providing Wnt ligands, BMP suppressant, and r-responsive proteins.

### 5.2. Paneth Cells

Results from studies of intestinal ISC derived from ISC indicate that the ISC niche can function independently in the absence of mesenchymal cells [41]. The most important component of this small intestinal crypt is the Paneth cells, which are located close to the ISC and provide niche signals that are essential for the survival and functioning of the ISC [11]. The antimicrobial peptides secreted by Paneth cells were found to form an essential part of the defense function of the ISC niche [42]. Moreover, Paneth cells express a variety of signaling molecules, including Wnt and Notch ligands, which are vital for the keep of the ISC niche [43]. While these studies only confirm the importance of these signals, the significance of Paneth cells in the ISC niche is critical. It was found that Atoh1 (also called Math1 and Hath1 in mouse and human separately) in the Notch signaling loop also exert a very important effect on Notch-based ISC destinies determination [44]. The data of the research suggested that in Atoh1^−/−^ mice, the absence of Paneth cells did not have an effect on the proliferation of ISC [45,46]. Therefore, it has been suggested that EGF and Wnt could serve as a replacement for Paneth cells and mediate Notch signaling to exert a vital effect on the keep of the ISC niche. However, knockdown of Sox9 or Gfli1 genes depletes Paneth cells, which in turn leads to the loss of ISC. Another study found that insertion of the diphtheria toxin receptor gene into the Reg4 locus in mice caused desiccation of the crypt basal cup cells [47,48,49]. In the large intestines, Reg4+ crypt basal goblet cells, in turn, can be regarded as a landmark of Paneth cell function, and depletion of this cell results in the decrease of ISC in the colonic crypts, which in turn harasses intestinal health and affects the growth of organoids [50]. However, it has been suggested that knockout mice are not an excellent model for studying whether Paneth cells exert a decisive effect on the functioning of the ISC niche, and the actual situation remains to be hoped that future trials will be carried out.

### 5.3. Enteroendocrine Cells (EECs)

Enteroendocrine cells also play an important role in the maintenance of intestinal homeostasis, one of the mechanisms of which is through the regulation of intestinal stem cell function. Intestinal stem cells act as rapidly circulating cells, which work together to maintain intestinal health by proliferating and differentiating to form different intestinal epithelial cells. Since they are located at the root of the intestinal crypts and only have access to a small supply of nutrients, intestinal stem cells are highly sensitive to their surrounding microenvironment and different diets, including high-fat diets and fasting [51,52]. After a few days on a high-fat diet, intestinal stem cells change their metabolic pathway from glycolysis to fatty acid-like oxidation, at which point intestinal stem cell proliferative activity increases [53]. Similarly, when mice were fasted, intestinal stem cell activity increased and their metabolism was predominantly fatty acid-like, suggesting the ability of intestinal stem cells to rapidly capture nutrient alterations in the microenvironment and respond accordingly [54]. Together, these results suggest that enteroendocrine cells can act as nutrient-sensing cells to transmit signals from the intestinal lumen to the microenvironment of intestinal stem cells in a timely manner to regulate intestinal stem cell activity.

Moreover, it was found that the lipid metabolism pathway of intestinal stem cells was upregulated in animals with insufficient enteroendocrine cells, which is consistent with the results obtained in the fasting model [55,56]. Meanwhile, enhanced mitochondrial activity and increased oxygen consumption in the intestinal crypts were also seen, demonstrating the role of enteroendocrine cells in the regulation of intestinal stem cells [55]. Together, these results suggest that enteroendocrine cells can influence the function of intestinal stem cells by regulating the metabolic activity of the intestinal crypts [56]. In addition, derivatives of enteroendocrine cells can also influence intestinal stem cell activity, and this is achieved through the combined action of multiple derivatives, whereas the effect of a single derivative is limited [57]. For example, on a standard chow diet that was as deficient in neurotensin as possible, the mice were still better tolerated and had no significant differences in total intestinal length, villus height, and crypt depth. However, under fasting conditions, cell proliferative activity and Wnt signaling in the distal small intestinal crypts were significantly reduced if neurotensin was lacking [58]. These results suggest that neurotensin can affect cell proliferative activity in the intestinal crypts by modulating Wnt signaling during nutritional deficiencies, further emphasizing the role of enteroendocrine cells in the regulation of intestinal stem cells.

In addition, enterochromaffin cells in the enteroendocrine cells can promote the proliferative activity of intestinal epithelial cells through their secretion of 5-HT [59]. Human organoid models from in vitro have also shown that 5-HT produced by enterochromaffin cells significantly promotes the proliferation of intestinal epithelial cells [60]. The results provide a disorder mechanism via which patients Cronkhite–Canada syndrome show up-regulated gut 5-HT level and gut polyposis [60]. Studies have shown that GLP-2 is one of the most effective derivatives secreted by enteroendocrine cells to regulate the proliferation of intestinal epithelial cells and that it has been used to ameliorate intestinal-related disorders such as short bowel syndrome [61]. Although GLP-2 is not required for intestinal development and homeostasis maintenance, exogenous supplementation with GLP-2 promotes crypt cell proliferation and increases villus and microvillus length [62]. This process is regulated by several complex mechanisms, including the Wnt signaling pathway [63,64,65]. Specifically, GLP-2 is used to promote the proliferative activity of intestinal stem cells by accelerating their entry into the S phase of the cell cycle. In addition, GLP-2 may act to nourish the gut [66,67]. Further studies showed that in Drosophila, EECs can regulate the proliferation of intestinal stem cells by signaling to cells within the subepithelial ecological niche [68,69].

## 6. Mechanisms of Intestinal Stem Cells Promoting Intestinal Mucosal Repair

Overall, the process of ISC to daughter cell transition is in a dynamic equilibrium. At the molecular level, multiple signaling pathways work in concert to facilitate intercellular communication and thus maintain intestinal homeostasis. Dysfunction of these signaling pathways, on the other hand, can lead to various intestinal diseases. Therefore, we next summarize several signaling pathways that exert a vital effect on the proliferation and differentiation of ISC.

### 6.1. Integrity of the Epithelium as a Barrier

The intestinal epithelial cell layer is made up of a combination of different types of cells, such as enterocytes, Paneth cells, absorptive cells, goblet cells, enteroendocrine cells, and microfold cells, which work together to maintain the homeostasis of the intestinal epithelial barrier [70,71]. Goblet cells exert defense and repair by secreting mucus, trehalose peptides, and resistin-like molecule-β. Enteroendocrine secretion not only senses nutrients and gut flora stimulation, but also delivers nutritional signals to the nervous system [72]. Paneth cells, in turn, provide intestinal stem cells with their essential growth factors to maintain the stemness of intestinal stem cells [11]. Paneth cells also secrete antimicrobial peptides including α-defensin and lysozyme C to clear bacteria [73]. Absorptive enterocytes, on the other hand, can play an immunological role by expressing polymeric immunoglobulin receptors (pIgR) that mediate IgA secretion by B cells [74] (Table 1).

#### 6.1.1. Mucus Layer

The mucus layer consists of a highly dynamic extracellular matrix located outside the intestinal epithelial cells and is considered the first mechanical barrier in the gastrointestinal tract. As a thicker gel-like substance, the mucus layer covers the surface of the gastrointestinal tract and separates the contents of the intestinal lumen from the intestinal epithelium and the cells beneath it [75,76]. This barrier effectively prevents harmful substances in the intestinal lumen from coming into direct contact with the intestinal epithelium and the cells beneath it, thus exerting a protective effect on the intestinal mucosa. The main component of mucus is MUC2, and they are interconnected to form a reticular structure [77]. The study by Bergstrom et al. revealed that colonic mucus contains two different O-glycosylated forms of Muc2 from the proximal and distal colon, and that they wrap fecal matter and microbiota tightly. Notably, some microbiota induce cup cells in the proximal colon to encapsulate themselves by producing MUC2. Not only that, MUC2 production in the proximal colon alters the structure and function of the microbiota [78]. The mucus layer not only separates the harmful flora, but also provides a place for beneficial bacteria. In addition, the network formed by the mucus layer traps and immobilizes pathogens, including bacteria and viruses, to prevent them from coming into direct contact with the epithelial cells [77]. The gut microbiota can also influence the mucus layer: it secretes enzymes that degrade mucins to obtain nutrients to support its growth [79]. However, the presence of sialic acid on mucin protects it from degradation by degrading enzymes secreted by the gut microbiota [80]. Thus, similar to the promising data achieved by targeting ECM proteins in tumors, therapeutics targeting mucins or their glycosylation may serve as an effective approach to prevent or ameliorate inflammatory bowel disease (IBD) [81,82].

#### 6.1.2. Antimicrobial Peptides

In the gastrointestinal tract, AMPs are mainly derived from epithelial cells, such as Pan cells and cup cells. Some AMPs from the gut exert their debacterizing effect by acting on the cell wall of bacteria [83,84]. The correlation between intestinal epithelial cells and AMPs is important for elucidating the functions and mechanisms of the body’s digestive system [85]. First, the intestinal epithelium acts as a guard, capable of recognizing pathogens and triggering a timely immune response. Immediately afterward, these cells initiate the secretion of AMPs, which in turn remove bacteria and viruses [83].

Of greatest interest in this process is the selective action of AMPs. This selectivity is critical for maintaining the dynamic balance of the gut flora. The homeostasis of the gut flora benefits from the interplay of harmful and beneficial bacteria, and if all bacteria were removed, this would be equally disruptive to the gut and even host health [84]. In addition, lysozyme secreted by Paneth cells, as a glycosidase, catalyzes the hydrolysis of the 1,4-β-glycosidic bond between the N-acetylglucosamine and N-acetylcytidylic acid components of the peptidoglycan, which in turn destroys the bacterial cell wall [86]. In addition, another antimicrobial peptide synthesized by Paneth cells can be utilized to remove bacteria by an enzymatic mechanism using its secreted phospholipase A2. These antimicrobial peptides include defensins, C-type lectins of the REG3 family and neutral endotoxin [87]. Most of these AMPs disrupt bacterial membranes, causing membrane potential collapse, loss of metabolites and ions, and osmotic lysis [88].

#### 6.1.3. Tight Junction

Tight junctions are key components that make up the intestinal epithelial barrier, and they seal the gaps between these specialized intestinal epithelial cells. The components that make up the tight junction structure include transmembrane proteins, articulin, and cytoskeleton [61,89]. Major participants include closure proteins, tight junction proteins, adhesion banding proteins, and closure banding proteins [90]. These proteins work in concert with each other to form a physical barrier that selectively regulates the entry and exit of molecules and ions. Thus, tight junctions primarily form a physical barrier between the intestinal lumen and the tissues of the body. This physical barrier is what prevents harmful substances, including pathogens, toxins, and undigested food particles, from entering the bloodstream [91]. In addition, this physical barrier is also involved in the nutrient absorption process, ensuring that adapted nutrients can be utilized by the body [91]. However, damage to the tight junctions can disrupt intestinal permeability, a phenomenon known as “leaky gut”. In this case, toxic and harmful substances take advantage of the opportunity to enter the body through the bloodstream, causing intestinal disorders and even systemic health problems [92].

### 6.2. BMP Signaling Pathway

A gradient of BMP signaling was observed along the crypt–villus axis, and BMP signaling could antagonize Wnt signaling and thus inhibit the renewal and proliferation of small ISC. This process plays a key role in inhibiting the over-activation of small ISC. In the small intestine of mice, overexpression of Noggin resulted in the development of a large number of neonatal crypts, whereas downregulation of Bmpr1a led to crypt hyperplasia [93]. It was shown that BMP signaling could antagonize the proliferation and renewal process of small ISC by directly preventing the Wnt signaling pathway [94]. Therefore, the knockdown of BMP in LGR5^+^ small ISC leads to rapid expansion of ISC, whereas inactivation of BMP signaling induces unrestricted expansion of LGR5^+^ small ISC, which in turn leads to small intestinal epithelial polyp development, a phenomenon observed in small intestines with radiation-induced inactivation of BMP signaling [95]. It was found that radiation induces BMP signaling inactivation, which in turn causes apoptosis in some LGR5^+^ small ISC, while healthy LGR5^+^ small ISC undergo damage repair [96]. The study emphasizes that if BMP signaling is inactivated at a young age [97], it can lead to other cells becoming desensitized to BMP signaling, ultimately resulting in malignant hyperplastic intestinal polyps [98]. However, it cannot be determined whether BMP signaling in epithelial or mesenchymal cells exerts a vital effect on inhibiting the growth of small intestinal polyps [99]. Although previous studies have used the overexpression of Noggin and knockout of Bmpr1a mouse models, they have not been able to resolve this issue [100].

### 6.3. Wnt/β-Catenin Signaling Pathway

Wnt signaling is progressively weakened along the small intestinal crypt–villus axis. Thus, the crypt region is an area of high Wnt signaling expression, which is also an area of high ISC density. In contrast, Wnt signaling intensity is relatively low in the villus region, which also corresponds to the remains of differentiated cells in this region. In the small intestine, Wnt signaling exerts a vital effect on the self-renewal and proliferation of small ISC. Study indicated that proliferating cells and ISCs were lost in large numbers in the small intestine of adult mice overexpressing Dkk1 [100] virus or specifically knocking out the Ctnnb1 (encoding β-catenin protein) gene, which in turn lead to the stopping of self-renewal of the entire intestinal epithelium [101]. In addition, Wnt overexpression leads to excessive proliferation of small intestinal crypt cells and expansion of stem cell regions, which increases the risk of colorectal cancer. The marker genes for intestinal stem and progenitor cells, LGR5 and Olfm4, are both modulated by Wnt [102]. Specifically, in crypts, Wnt molecules secreted by epithelial or stromal cells first bind to LRP5/6 receptors, which in turn activate β-catenin and upregulate its expression, followed by binding of β-catenin to the nuclear transcription factor TCF4, which ultimately maintains stem cell stemness and promotes their proliferation and differentiation [103]. However, suppression of the Wnt/β-catenin signaling loop hinders the proliferation of ISC. It was shown that knockdown of the TCF4 gene in mouse embryonic intestinal epithelial cells lead to stagnant proliferation of small intestinal villus cells in newborn mice. Similarly, knockdown of TCF4 with Ctnnb1 in the intestinal epithelial cells of adult mice caused blocked cell proliferation in the crypt region of mice [104]. Therefore, Wnt signaling exerts a vital effect on stemness maintenance, proliferation and differentiation of ISC [105].

### 6.4. Notch Signaling Pathway

In chickens, the mechanism by which the Notch signaling loop mediates intestinal stem cell differentiation has not been investigated and can only be obtained by comparison with mammals. The Notch signaling pathway exists in almost all postnatal animals and is a closely conserved signaling mechanism that functions by cell–cell communication [106]. Within the small intestinal epithelium, the several ligands and receptors for Notch are expressed only in the crypts, and thus their activity exists only in that region [107]. Notch signaling exerts a vital effect on regulating the proliferation, differentiation, and self-renewal of small ISC, especially during the differentiation stage of small ISC [108]. Activation of the Notch signaling loop induces the differentiation of stem cells to absorptive cells and inhibits their differentiation to secretory cells [109]. Moreover, Notch signaling also plays an important role in modulating the renewal process of small ISC. In the microenvironment of ISC, Paneth cells can provide ligands for Notch signaling to be expressed abundantly in this microenvironment [110]. Lineage tracing trials have clarified that small ISC are a subset of cells characterized by high Notch signaling, and that this subset of cells has a unique ability to differentiate and give rise to all of the different cell types found in intestinal epithelial cells [111].

### 6.5. Hedgehog Signaling Pathway

Hedgehog (Hh) signaling is primarily vital during host embryonic development, which in turn continues to influence proliferation and differentiation after cytogenesis [112]. Three family members of Hh exist in mammals: the Sonic Hh, Indian Hh (Ihh), and Desert Hh. Ihh is the predominantly present Hh protein in the gut and mediates paracrine signaling initiated by differentiated epithelial cells to regulate mesenchymal cells. Notably, Ihh also inhibits the proliferation of columnar cells in the crypt by activating BMP signaling [113]. Not only that, Ihh has the ability to negatively regulate immune function within the lamina propria. Knockdown of the Ihh gene activates an immune process similar to the wound healing process, which involves remodeling of epithelial cells and recruitment of fibroblasts and macrophages, a process that does not damage the epithelial cells themselves [114]. Thus, reduced expression of Ihh in epithelial cell injury or dysfunction may be one of the important mechanisms for activating the Leymic cells wound healing response [115].

### 6.6. EGF Signaling Pathway

EGF receptors, such as EGFR, ErbB-2, ErbB-3, and ErbB-4, are collectively referred to as the receptor complex kinase family, and these receptors play important roles in cellular signaling transduction. They first bind to the growth factor EGF and subsequently activate range of downstream signaling loops, including PI3K/Akt, PLCγ/PKC, and Ras/Raf/Mek/Erk signaling loops, etc., which ultimately control cell proliferation and differentiation, especially of small intestinal ISCs and TA cells [116,117]. The downstream Ras/Raf/Mek/Erk signaling loop of EGF is a region of crypt a particularly active region [118]. To combat the over-activation of small ISC, small intestinal epithelial cells have also derived a series of negative feedback regulatory mechanisms to antagonize the EGF signaling pathway. Among them, Lrig1, as one of the many negative feedback regulators of EGF signaling, is mostly expressed in proliferating cells in the crypts of the small intestine. Specific knockdown of the Lrig1 gene in the small intestine induces expansion of the proliferative zone of the crypts, which in turn induces small bowel tumorigenesis [118,119].

## 7. Hippo Signaling Pathway

Hippo signaling is a highly conserved cascade signaling mechanism commonly found in drosophila and mammals, which participates in several biological processes, including cell proliferation, differentiation, survival, and ultimately determines cell fate and maintains tissue homeostasis, by regulating its key target genes [120,121]. In contrast, an aberrant Hippo pathway induces the onset and development of cancer or other diseases, in which it participates in the process of tumor formation via modulating cell proliferation and apoptosis [122]. In addition, blocking the nuclear translocation of YES-associated protein (YAP) and PDZ-binding motif (TAZ) inhibits tumor development by reducing cell proliferation and increasing apoptosis [123].

Not only that, the Hippo signaling loop also exerts a vital effect on keeping intestinal health, as evidenced by the fact that inhibition of the Hippo signaling loop results in organism overgrowth and induces tumorigenesis. When MST1 and MST2 were specifically knocked down in intestinal epithelial cells, the villus structure of mice was homeostatic and showed expanded undifferentiated cells [124]. Not only that, abnormal epithelium and adenomas develop in the colon of these mice. Similarly, the small and large intestine in sav1 gene-deficient mice also exhibit expanded crypt structures with increased polyp formation. However, a number of studies have found that the gut does not show adverse phenotypes when depleted of YAP1 and/or TAZ protein expression in the gut in the healthy state, suggesting that the importance of YAP1 and TAZ is not emphasized in the healthy state [125,126,127]. In contrast, MST1, MST2, and SAV1 in the Hippo pathway are essential for healthy intestinal tissue [126]. Overall, an impaired Hippo loop results in over-activation of YAP1 and TAZ, resulting in lost ISCs proliferation and increased tumorigenesis. The current study was only conducted in mice with gut-given knockout of MST1, MST2, SAV1, YAP1, and/or TAZ, and more studies are needed to corroborate this.

It has been shown that YAP1 and TAZ can maintain and promote ISCs activity in several organs not limited to the gut, including the liver, skin, and nerves [128]. In the intestine, the endogenous YAP1 protein is expressed only in the ISCs ecological niche at the base of the crypt, implying that he may be associated with the regulation of ISC activity [129]. It has been found that highly active YAP1 promotes the proliferation of ISC [126]. Overexpression of YAP1 in the intestine results in expansion of undifferentiated cells and self-renewal of differentiated cells present at the tip of the villi, a phenomenon that is similar to the phenotype of intestines lacking MST1 and MST2 genes. In addition, intestinal gene transfer techniques revealed that YAP1 with TAZ promotes the binding of TEAD transcription factors to stem and progenitor cells and further causes progenitor cells to differentiate into cup cells [130]. In the healthy state, the tumor suppressor gene protein kinase C zeta type (PKCζ) in ISC can directly inhibit YAP1 activity by phosphorylation [131]. Universally, active expression of YAP1 and TAZ promotes ISC activity. However, it was found that when YAP1 expression was specifically induced in the mouse intestine, it disrupted the normal intestinal tissue structure, resulting in phenotypes such as mislocalization of Paneth cells or reduction in the number of ISC, and ultimately inhibited the proliferation of ISC in the crypt [127]. This result indicates that YAP1 functions as a growth inhibitor in the balanced intestine. The reason for the inconsistency of this result with previous results may lie in the difference between the experimental setup and the mouse model [127]. Specifically, researches suggested that mice with inducible systemic overexpression of YAP1 were used, whereas Barry specifically induced YAP1 expression in intestinal epithelial cells. Intestinal epithelial cells continuously work with their neighboring stromal and immune cells, and in the process can modulate YAP1 activity through chemical or physical pathways. Thus, the different phenotypes detected in different models may be due to autonomous and non-autonomous influences. In the nucleus, YAP1 activates the Wnt signaling loop, whereas in the cytoplasm, YAP1 inhibits the Wnt signaling pathway, both of which can have opposite effects on the maintenance and proliferation of progenitor cells. In addition, YAP1 can also initiate a negative feedback regulatory mechanism via causing the expression of LATS2, which in turn acts as a growth inhibitor [132,133,134].

**Figure 4:**
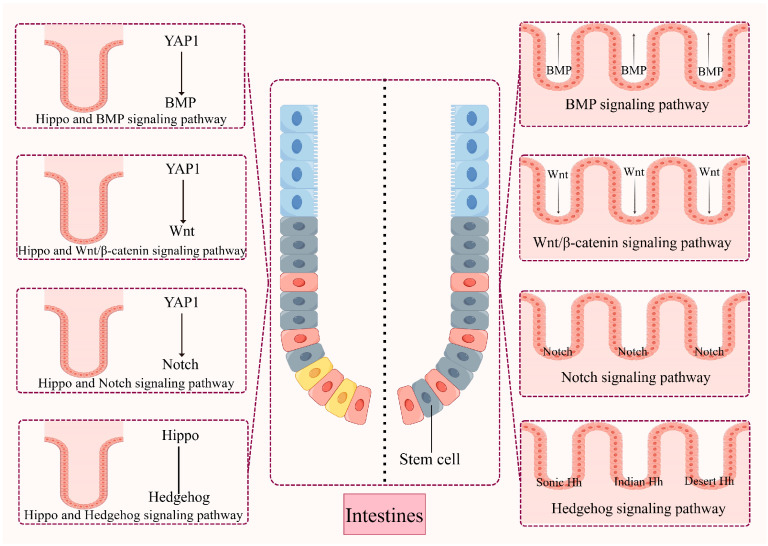
Potential mechanisms for regulating intestinal stem cells. Intestinal stem cell-regulated intestinal epithelial homeostasis is tightly controlled and regulated by interrelated developmental pathways that keep cell proliferation and differentiation processes in balance. Several conserved signaling pathways, such as the Wnt, Notch, hedgehog, and bone morphogenetic protein pathways, have been shown to regulate intestinal homeostasis and regeneration in mammals. The Hippo signaling system, as an exceptionally well-preserved cascade signaling mechanism, can play key roles in cell proliferation, cell survival, cell differentiation, cell fate determination, organ size, and tissue homeostasis by interacting with these signaling pathways.

Intestinal epithelial balance is closely controlled and modulated by interrelated developmental loops, allowing the cell proliferation and differentiation processes to remain relatively balanced. Signaling loops such as Wnt, Notch, Hh, and BMP have been indicated to exert a vital effect on maintaining mammalian intestinal homeostasis and regeneration by interacting with the Hippo pathway [135]. Understanding how the Hippo pathway interacts with these signaling loops to regulate intestinal homeostasis will help develop appropriate therapeutic strategies (Figure 4).

### 7.1. Hippo and Wnt Signalling

Wnt signaling exerts a vital effect on the keep of gut homeostasis and stem cell activity [136]. The classical Wnt signaling pathway uses β-catenin as its essential inductor, and β-catenin together with the TCF and LEF families as transcription factors, which in turn trigger the transcription of marker genes [137]. Nuclear localization of β-catenin has been observed in mouse ISC located at the base of the crypt, which represents active Wnt signaling [138]. Thus, in mice, suppression of classical Wnt signaling results in loss of transporter-expanded cells and damage to the crypt structure, and further leads to an imbalance of homeostasis and associated dysfunction in the intestine [124]. The interplay between the Hippo pathway and the Wnt signaling pathway has been intensively investigated in the context of keep of ISC activity and damage restoration. Our results indicate that YAP1 can maintain ISC activity and promote stem cell proliferation in the mammalian intestine via activating the Wnt signaling loop [124].

Specifically, overexpression of YAP1 induced the accumulation of β-catenin and the expression of its target genes in the nucleus. Moreover, the expression of Wnt target genes and ISCs markers Lgr5 and Ascl2 was significantly increased in the intestines of mice specifically knocked down for MST1 versus MST2 genes [125]. In vitro findings showed that down-regulation of YAP1 expression in the SW480 human colon cancer cell line further resulted in decreased transcription of β-catenin [125]. Overall, YAP1 expression upregulated Wnt signaling activity. In addition, it has also been shown that YAP1 and TAZ in the cytoplasm can lead to restriction of classical Wnt signaling, and many regulatory mechanisms have been suggested to reveal this modulation based on the current findings. First, YAP1 and TAZ in the cytoplasm can bind to disheveled homologue DVL-1 (be famed as DVL), a positive modulator of Wnt, and further inhibit DVL expression [139]. In addition, YAP1 with TAZ can directly inhibit nuclear translocation of β-catenin; secondly, YAP1 with TAZ can join in a complex that destroys β-catenin and recruits F-box/WD repeat protein 1A (also known as β-transducing repeat protein, β-TrCP), which ubiquitinates β-catenin and results in its degradation. In the mouse intestine, overexpression of YAP1 means that there is a concomitant increase in cytoplasmic levels of YAP1, which in turn further reduces the expression of β-catenin and its target genes [127]. However, many researches have indicated that YAP1 and TAZ can also be inversely triggered by Wnt signaling and that this is still essential for a number of Wnt responses in the intestine. In particular, the non-classical Wnt signaling pathway, which has also been found to play a crucial role in intestinal tissue regeneration, is a process that is independent of β-catenin [129,140,141]. In addition, Wnt signaling of the nonclassical pathway can activate YAP1 and TAZ signaling, which in turn can inhibit the typical Wnt signaling pathway in vitro [142]. Therefore, it remains unclear whether the nonclassical Wnt signaling loop is mediated by YAP1 and Wnt signaling during intestinal regeneration [143]. The crosstalk between Hippo and Wnt signaling loops is complicated and interesting and will need to be explored in depth in future studies.

### 7.2. Hippo and Notch Signalling

Apart from the Wnt signaling pathway, the Notch pathway is also involved in maintaining ISC activity and proliferation and differentiation. There are four Notch receptors in mammals, including Notch1/2/3/4, each of which contains range of extracellular ligand-binding structural domain, a transmembrane structural domain, and an intracellular structural domain. Upon activation of the ligand, the Notch intracellular structural domain (NICD) is dissociated from the receptor and translocated to the nucleus [144]. Subsequently, the nuclear NICD binds to the recombining binding protein suppressor of hairless (RBPJ, also regarded as CSL (CBF1/RBPjκ/Su(H)/Lag-1)) and induces the expression of target genes including HES transcription factors and others. In mice, inhibition of Notch expression further leads to a down-regulation in the expression of Lgr5 and olfactomedin 4 (Olfm4) and the ISCs number located at the bottoms of the crypt [145]. In addition to maintaining stem cell activity, Notch is also associated with the fate decision of stem cell differentiation. High Notch activity significantly accelerates the differentiation of transit-expanded cells, whereas suppression of Notch signaling leads to loss of ISCs activity [44]. Notably, the Notch signaling pathway is also modulated by the Hippo pathway [124]. The results showed that specific knockdown of MST1 and MST2 genes in the intestine resulted in increased nuclear localization of NICD and upregulation of Hes1 gene expression in the intestine [146]. In contrast, suppression of Notch signaling attenuated the extension of undifferentiated intestinal progenitor cells caused by YAP1 overexpression [125]. Collectively, these findings indicate that YAP1 positively regulates Notch signaling in the intestine and that YAP1 induces the expansion of ISC by mediating Notch. Although how Hippo regulates Notch is unknown, some members of the Notch family, including Notch1/2/9, and Hes1, were up-regulated in YAP1-overexpressing hepatocytes, and Notch2 was regarded as a direct marker gene of the YAP1-tead complex [147]. The phenomenon may offer a theoretical reference for a deeper understanding of the interaction mechanism between Hippo and Notch in the intestine.

Notch signaling regulates intestinal health by controlling ISCs and inducing absorptive cells differentiation. Active Notch stimulates differentiation of TA cells along the uptake spectrum [148], whereas low non-active Notch results in dysfunction of stem cell properties and inducing differentiation of the secretory spectrum [149]. In addition, the Hippo pathway also regulates Notch signaling. If Notch signaling is positively regulated by YAP1, YAP1 can mediate Notch to promote the amplification of ISCs [150,151]. Hippo and Notch signaling loops cause the secretion of target genes, including *Wnt5a* and *Wnt9b* [124]. The Wnts associated with many Frizzled receptors to stimulate Wnt signaling in ISCs, and ultimately, the proliferation and differentiation activities of ISCs were significantly enhanced. Interaction of Notch signaling, Notch1, and Dll1 between Paneth cells and ISCs regulates small intestinal health by controlling ISC and inducing absorptive cells [152]. It is also clear that Notch may not directly regulate the differentiation of small intestinal epithelial cells through the Hippo signaling pathway. Yet the molecular mechanisms by which the Hippo pathway modulates intestinal Notch signaling remain unclear. Our overview offers understanding into the interplay between Hippo and Notch signaling loops in regulating ISCs activity. Some degree of intestinal epithelial regeneration is effective in improving IBD. YAP1 modulation has been regarded as an effective mechanism for intestinal epithelial regeneration in IBD in DSS-caused colitis mouse model [153]. The finding of nAChRs in non-neuronal cells and their effect on non-nervous disorder, such as IBD is indispensable for researching treatments [154]. It was concluded that a three-part mechanism of nAChR, Hippo, and Notch signaling would allow for revision of the rate of ISC and its daughter cells to keep ISCs and daughter cell density in adult mice over time.

### 7.3. Hippo and BMP Signalling

BMPs belong to the transforming growth factor β (TGF-β) superfamily, and they bind first to mitogen-activated protein kinase receptors and then through activation of Smad-dependent or non-dependent pathways [155]. BMP signaling plays a key role in embryonic development and adult stem cell homeostasis by regulating cell proliferation, differentiation and apoptosis, and its dysfunction induces diseases [156]. BMPs are secreted proteins that bind to BMP receptors (BMPRs) as homodimers or heterodimers. BMP-2/4 are the major BMPs present in the gut [157]. The BMP signaling pathway plays a crucial role in gut development and homeostasis maintenance from Drosophila to vertebrates. In Drosophila, Bmp-2/4 homologs Dpp and Gbb are essential for midgut development, differentiation, and regeneration [158]. BMP and its receptors are expressed in both epithelial and mesenchymal cells of the mammalian intestine, suggesting that there is an exchange of information between the epithelium and the mesenchyme, but the signaling of BMP in them is complex [159]. In addition, BMP activity is modulated by the antagonists, including Noggin and Gremlin 1, which are mainly expressed in mesenchymal cells around the crypt, and their aberrant expression has been associated with colon tumorigenesis. Through transmembrane receptors, BMP could trigger responses via Smad or non-Smad pathways.

Signal communication between Hippo and BMP or Hh has also been studied and confirmed, although this study was not conducted in intestinal cells. Studies have demonstrated that BMP exerts a vital effect on regulating cell differentiation, proliferation and growth [160]. In the intestine, inhibition of the BMP signaling loop using Noggin, an antagonist that overexpresses BMP, resulted in the overproliferation of ISC, an up-regulation in the crypts number, and ultimately the induction of intestinal polyposis. The data indicate that BMP negatively modulates the proliferation of ISC in intestinal crypts [93,94]. Activation of the BMP signaling loop occurs first with the binding of BMP ligands to type I and type II serine/threonine kinase receptors, followed by phosphorylation of the C-terminus of the activated receptors on SMADs (SMAD1, SMAD1, SMAD5, and SMAD8), which then binds to SMAD4 and ultimately translocates to the nucleus. The transcriptional complexes of SMAD1, SMAD5, SMAD7, and SMAD4 are completely activated by phosphorylation of the linker region of cyclin-dependent kinase (CDK)8 and/or CDK9. The ISCs in mouse embryo, YAP1 is recruited by the phosphorylated SMAD1-SMAD4 complex to the accelerator regions of BMP-responsive genes, thereby inducing the expression of BMP target genes [161].

### 7.4. Hippo and Hedgehog Signalling

The Hh signaling pathway, which is closely related to intestinal homeostasis, has been described previously, and Indian hedgehog and Sonic hedgehog, two important Hh signaling proteins, have been shown to be expressed in the intestinal epithelium of mice [162]. It was found that depletion of Indian hedgehog in the mouse small intestines resulted in an overexpansion of the ecological niche of ISC and a reduction in ISCs differentiation [163]. Although signaling crosstalk between the Hippo pathway and the Hh pathway in the intestine has not been identified, it has been demonstrated in other organs. In medulloblastoma, where the tumor originates from cerebellar granule neuron precursors (CGNPs), sonic hedgehog signaling promotes their expression [164,165]. Not only that, activation of sonic hedgehog signaling in CGNPs also upregulates the protein level of YAP1, which directly binds to insulin receptor substrate 1 (IRS1), which in turn leads to nuclear localization of YAP1 [166]. Moreover, YAP1 deficiency counteracts sonic hedgehog-induced proliferation of CGNP. sonic hedgehog signaling downstream of the factor GLI2, which has been regarded as a target gene of the YAP1–tead complex, suggests a mechanism by which YAP1 drives adult neural tube cell tumorigenesis. In the intestine, however, the predominant Hh protein is Indian hedgehog. therefore, crosstalk between Hh and Hippo signaling pathways in the intestine may be controlled by Indian hedgehog, but this needs to be further explored. Overall, the Hippo signaling loop has been suggested to crosstalk with the BMP and Hh loops, and one could hypothesize that this crosstalk also exists in the intestine, which in turn maintains intestinal homeostasis.

In general, hedgehog signaling modulates many organs’ differentiation, proliferation, and morphogenesis, including patched (PTCH) and smoothened (SMO) trans membrane proteins. Apart from this, the loops include Indian hedgehog (IHH), desert hedgehog (DHH), Sonic hedgehog (Shh), suppressor of fused protein (SUFU), and glioma-associated oncogene (GLI) transcription factors. Dysfunction of this signaling pathway has been reported to be associated with a variety of cancers. Binding of ligand Hh to the PTCH receptor further internalizes and degrades the receptor protein, which in turn releases SMO and leads to dissociation of the SUFU-GLI complex. Ultimately, GLI transcriptional activators translocate to the nucleus and induce the expression of hedgehog target genes including GLI1 and PTCH. In addition, in the absence of ligand, PTCH hinders SMO expression and causes pathway blockage [167].

Studies have shown that abnormalities in the Hh signaling pathway are associated with the development of several intestinal cancers [168]. Dysfunctional Hh signaling pathways can be classified into three types, each of which can induce different types of cancers separately: autonomous and ligand-independent types of Hh signaling, ligand-dependent oncogenic Hh signaling in autocrine/juxtacrine manner, and ligand-dependent Hh signaling in paracrine or reverse paracrine manner [169]. Mutations often result in non-dependent ligand activation. Either SMO mutations (activation) or PTCH mutations (inactivation) activate the Hh signaling pathway, which in turn promotes tumor growth [170]. During autocrine/paracrine signaling, similar tumor cells produce and take up a ligand. Overactivation of Hh ligands promotes tumor growth during the pathogenesis of several cancers [171]. Studies have shown that paracrine activation of the Hh pathway can exacerbate cancer by binding to PTCH receptors on tumor cells, leading to increased proliferation and differentiation [172].

The Hippo pathway, a highly conserved pathway, can regulate biological processes including cell proliferation and differentiation by activating target genes, and dysregulation of this pathway induces the development of a variety of intestinal diseases. The Hippo pathway is composed of mammalian Ste20-like kinases 1/2, large tumor suppressor 1/2, yes association protein, and its paralog transcriptional coactivator with PDZ-binding motif (TAZ) (also called be WWTR1) [173]. Activation of the Hippo pathway induces phosphorylation of MST1/2, which further activates LATS1/2 and inhibits YAP/TAZ activity [174]. Subsequently, YAP/TAZ is confined to the cytoplasm and degraded by the proteasome, which further triggers signaling pathway dysregulation that ultimately promotes cancer progression [175].

## 8. Concluding Remarks and Future Perspectives

Multifunctional intestinal epithelial cells have organized heterogeneity. The single layer of epithelial cells is responsible for the digestion and absorption of nutrients and the secretion of digestive enzymes and other enzymes and regarded as a protective barrier stopping harmful substances. Chickens are precocious animals; thus, the intestines are thoroughly matured in the postembryonic stage. Several basic studies over the past decades have examined chicken embryonic gut evolution. Yet, the detailed cytoarchitecture and regulatory mechanisms of chicken intestinal epithelial cell differentiation are currently unknown. In this paper, we present detailed knowledge of chicken intestinal epithelial dynamics and physiology by describing the intestinal developmental process, differentiation, modulation, and cellular dynamics. In addition, we also have similarities and differences between birds and mammals. Finally, we concisely describe recent in vitro models for the detection of chicken small intestinal epithelium, in particular to offer new ways for future studies of the molecular signaling pathways that regulate cell proliferation and differentiation of chicken ISCs.

This review offers a basis for further study of the influences of pathogen incidence and nutritive supplementation on the differentiation or development of the small intestinal epithelium in chickens. Based on the prior literature, chickens were observed to differ from mammals and rodents in (1) the proliferation of intestinal epithelial cells from the crypt villi, and (2) the exists of Paneth cells that are controversial and may appear in both crypts and villi. These results may indicate that there are distinct underlying mechanisms for the differentiation and proliferation of chicken intestinal epithelial cells. Therefore, it is important to conduct further studies to understand the unique knowledge of the chicken intestinal epithelium so as to correctly analyze the nutritional or microbial connections that influence the health of the chicken intestinal epithelium.

## Figures and Tables

**Table 1:** Different intestinal epithelial cells and their functions. Intestinal epithelial cells include goblet cells, EECs, microfold cells, Paneth cells, and absorptive enterocytes. They function to protect the gastrointestinal tract through different pathways of action.

Cell Type	Mechanism of Action	Functionality
Goblet cells	Mucus trefoil peptides, and resistin-like molecule-β [70]	crucial for epithelial defense and repair
EECs	Triggers secretion of peptide or amine hormones	sense nutrients and microbial stimuli, transmitting nutritional information to the nervous system [72]
Microfold cells	IgA	presenting bacterial antigens to dendritic cells [46,74]
Paneth cells	secreting antimicrobial peptides, such as α-defensins, lysozyme C, regenerating islet-derived protein IIIβ (RegIIIβ), regenerating islet-derived protein IIIγ, and angiopoietin-4	support stemness in the intestinal stem cell niche by providing essential growth factors to cryptbasal columnar stem cells [75]
Absorptive enterocytes	express polymerized immunoglobulin receptors (pIgR) and transmembrane mucins, forming a glycocalyx	pIgR mediates the transcellular transport of IgA released by B cells from the basolateral to the apical surface and to the glycosyl surface facing the lamina propria [76]

## Data Availability

Data will be made available on request.

## Abbreviations

ISC	Intestinal stem cells
TA	Transport amplification
BrdU	5-bromo-2-deoxyuridine
PCNA	Proliferating cell nuclear antigen
IBD	Inflammatory bowel disease
ECM	Extracellular matrix
EGF	Epidermal growth factor
BMP	Bone morphogenetic proteins
MSCs	Mesenchymal stem cells
Hh	Hedgehog
Ihh	Indian Hh
YAP	YES-associated protein
TAZ	PDZ-binding motif
PKCζ	Protein kinase C zeta type
NICD	Notch intracellular structural domain
Olfm4	Olfactomedin 4
CDK	Cyclin-dependent kinase
CGNPs	Cerebellar granule neuron precursors
IRS1	Insulin receptor substrate 1
